# Functional redundancy of *Burkholderia pseudomallei* phospholipase C enzymes and their role in virulence

**DOI:** 10.1038/s41598-020-76186-z

**Published:** 2020-11-06

**Authors:** Varintip Srinon, Patoo Withatanung, Somjit Chaiwattanarungruengpaisan, Metawee Thongdee, Chatruthai Meethai, Joanne M. Stevens, Richard W. Titball, Sunee Korbsrisate

**Affiliations:** 1 Department of Immunology, Faculty of Medicine Siriraj Hospital, Mahidol University, Bangkok, Thailand; 2 Veterinary Diagnostic Center, Faculty of Veterinary Science, Mahidol University, Nakhon Pathom, Thailand; 3 The Monitoring Surveillance Center for Zoonotic Diseases in Wildlife and Exotic Animals, Faculty of Veterinary Science, Mahidol University, Nakhon Pathom, Thailand; 4 Division of Infection and Immunity, The Roslin Institute and Royal (Dick) School of Veterinary Studies, University of Edinburgh, Midlothian, UK; 5 Department of Biosciences, University of Exeter, Exeter, UK

**Keywords:** Immunology, Microbiology

## Abstract

Phospholipase C (PLC) enzymes are key virulence factors in several pathogenic bacteria. *Burkholderia pseudomallei*, the causative agent of melioidosis, possesses at least three *plc* genes (*plc1*, *plc2* and *plc3*). We found that in culture medium *plc1* gene expression increased with increasing pH, whilst expression of the *plc3* gene was pH (4.5 to 9.0) independent. Expression of the *plc2* gene was not detected in culture medium. All three *plc* genes were expressed during macrophage infection by *B. pseudomallei* K96243. Comparing *B. pseudomallei* wild-type with *plc* mutants revealed that *plc2, plc12* or *plc123* mutants showed reduced intracellular survival in macrophages and reduced plaque formation in HeLa cells. However, *plc1* or *plc3* mutants showed no significant differences in plaque formation compared to wild-type bacteria. These findings suggest that Plc2, but not Plc1 or Plc3 are required for infection of host cells. In *Galleria mellonella*, *plc1, plc2* or *plc3* mutants were not attenuated compared to the wild-type strain, but multiple *plc* mutants showed reduced virulence. These findings indicate functional redundancy of the *B. pseudomallei* phospholipases in virulence.

## Introduction

*Burkholderia pseudomallei*, a Gram-negative facultative intracellular bacterium, is the etiological agent of melioidosis in humans and in animals. Melioidosis in humans was at one time thought to be largely restricted to Southeast Asia and Northern Australia, but it is now thought to occur in many tropical and sub-tropical regions of the world^1^. The annual global burden of meliodosis is estimated to be 165,000 cases with 89,000 deaths from the disease^1^. A feature of *B. pseudomallei* is its ability to modulate a range of host-cell responses and to evade phagocyte killing activity^2,3^. *B. pseudomallei* has evolved mechanisms to evade phagocyte activities, including escape from phagosomes and entry into host cell cytosol where it multiplies and forms actin tails allowing cell-to-cell spreading^4^. This complex intracellular lifestyle is contributed by several bacterial virulence factors including type three secretion systems (T3SS), type six secretion systems (T6SS), polysaccharide capsule, lipopolysaccharide (LPS), and various secreted effector proteins^5^. Additionally, *B. pseudomallei* can produce many enzymes which play roles in virulence including proteases, catalase, peroxidase, superoxide dismutase^1^, and phospholipase C (Plc) enzymes^6^. Plc enzymes play roles in the pathogenesis of several Gram-positive and Gram-negative bacterial infections including those caused by *Mycobacterium tuberculosis*^7^, *Pseudomonas aeruginosa*^8^, *Clostridium perfringens*^9^, *Listeria monocytogenes*^10^, and *Legionella pneumophila*^11^. Also, different of Plcs play different roles in virulence^12^, including tissue colonization, evasion of host defense mechanisms, escape from host cell phagosomes and/or the induction of mediators of inflammation^13^.

Analysis of the *B. pseudomallei* K96243 genome reveals genes encoding three Plc enzymes (Plc1, Plc2 and Plc3). The genes encoding Plc1 (*bpsl2403*) and Plc2 (*bpsl0338*) are located on chromosome 1. These encoded proteins are predicted to be acidic, have the ability to hydrolyze phospholipids including phosphatidylcholine and sphingomyelin and are non-hemolytic^14^. The gene encoding Plc3 (*bpss0067*) is located on chromosome 2^5^. At present, the conditions under which the *plc* genes are expressed, and their roles in virulence are poorly understood. We have previously characterized the *B. pseudomallei* Plc1 and Plc2 and found that Plc2 was cytotoxic^14^. Additionally, these Plc enzymes appear to play a role in nutrient acquisition^14^. Subsequently, Burtnick et al*.*^15^ showed that *B. pseudomallei* Plc1 and Plc2 are secreted from the bacterial cell via the type II secretion system (T2SS) in a GspD-dependent manner^15^.

Little is known about the *B. pseudomallei* Plc3 enzyme. Whole-genome microarrays have revealed that *plc3* is up-regulated in vivo, and in hamsters a *plc3* mutant shows reduced virulence compared to the wild-type, suggesting that it is required for virulence^16^. However, the mechanisms underlying attenuation are unknown. Dowling et al*.*^17^ reported that Plc3 might be a potential candidate vaccine requiring further study.

In this study, the expression of the *plc* genes in culture medium and in J774A.1 macrophage-like cells was analyzed using RT-PCR. *B. pseudomallei plc1*, *plc2* or *plc3* single mutants and *plc12* or *plc123* mutants were assessed for virulence in macrophages and in *Galleria mellonella* larvae. Our work provides new insights into the role of Plc enzymes in the pathogenesis of disease caused by *B. pseudomallei*.

## Results and discussion

### Culture pH differentially affects *plc1* gene expression

**Figure 1:**
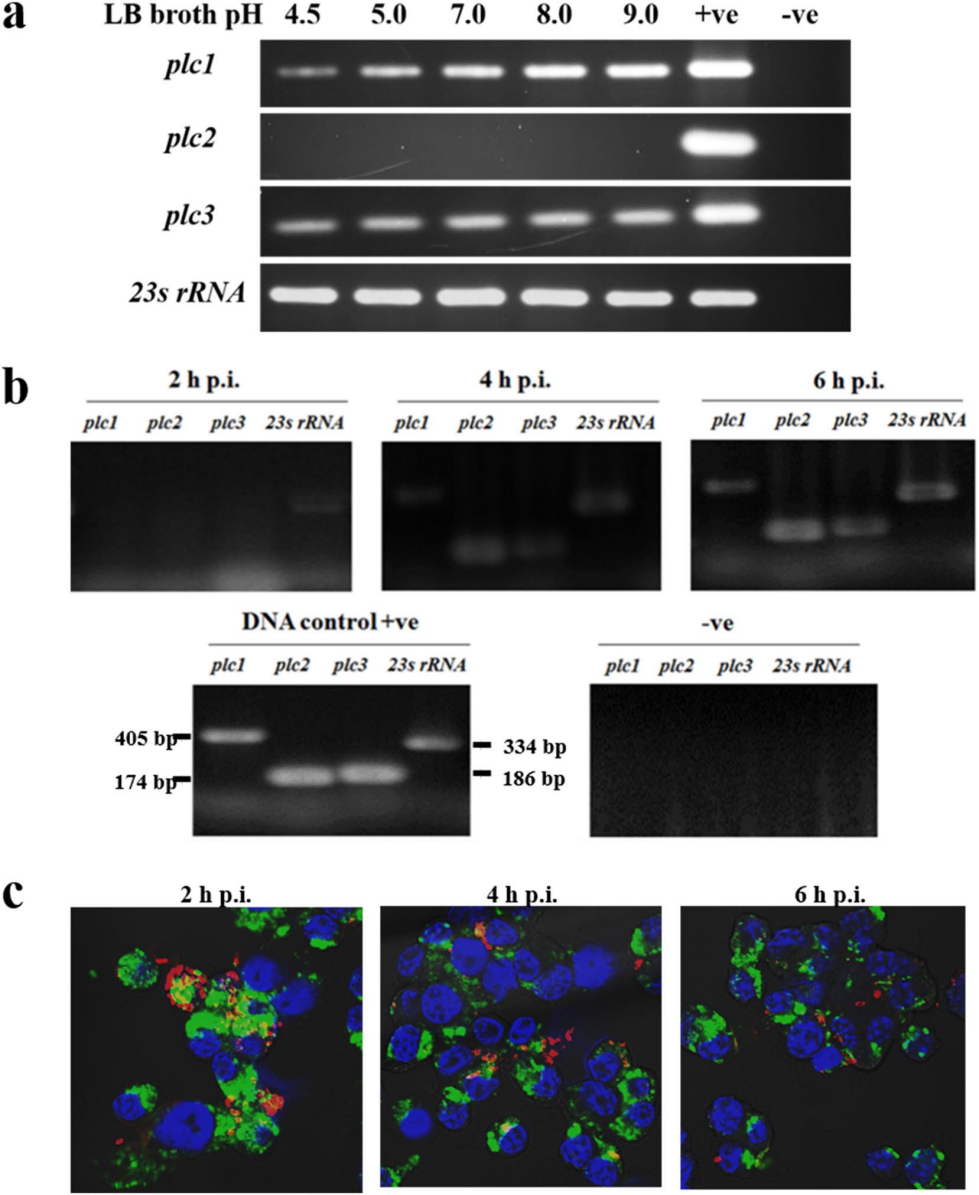
Reverse transcription (RT)-PCR analysis of *B. pseudomallei plc* expression. (**a**) Expression of *plc* genes in LB broth adjusted to pH 4.5, 5, 7, 8 or 9. *B. pseudomallei* wild-type K96243 was incubated for 15 min in LB broth at the pH indicated before RNA extraction. Positive control (+ ve) was *B. pseudomallei* genomic DNA. DNase-treated bacterial RNA was used as a negative control (− ve) to confirm the absence of DNA contamination in RNA samples (full-length gels are presented in Supplementary Fig. 1). (**b**) Expression of *plc* genes in J774A.1 macrophage-like cells infected with *B. pseudomallei*. RNA of *B. pseudomallei* K96243 was harvested from infected macrophages at 2, 4 or 6 h p.i. and converted to cDNA for PCR analysis with primers specific to each *plc* gene. Positive control (+ ve) was *B. pseudomallei* genomic DNA. DNase-treated bacterial RNA was used as a negative control (−ve). Unfortunately we are not able to reproduce the full length image of this gel because this image file was inadvertently deleted after this project was completed and before we submitted this manuscript. (**c**) Confocal micrographs of J774A.1 macrophages infected with *B. pseudomallei* (MOI 10) showed that majority of *B. pseudomallei* were within phagosomes at 2 h post-infection (p.i.). Escape of bacteria from the vacuoles was first observed at 4 h p.i., and most bacteria were within the cytosol at 6 h p.i.

*B. pseudomallei* is an intracellular bacterium*.* After phagocytosis by phagocytes, the bacteria encounter acidic condition within the phagosome, before escaping to survive in the cytosol^4^. To determine the effect of pH on the expression of the *plc1-3* genes, *B. pseudomallei* K96243 was incubated in LB broth which had been adjusted to pH 4.5, 5.0, 7.0, 8.0 or 9.0 before bacterial mRNA was extracted and tested. Using RT-PCR we first showed that expression of *B. pseudomallei* 23S rRNA was similar at all pH conditions tested. RT-PCR revealed that the level of transcription of the *plc1* gene increased between pH 4.5 and 9.0. This result suggests that the pH of bacterial cultures can differentially affect *B. pseudomallei plc1* gene expression. In contrast, the level of expression of *plc3* was similar at all pH values tested. Expression of the *plc2* gene was not detected under any of the conditions tested (Fig. 1a) (full-length gels are presented in Supplementary Fig. 1). Our finding that *plc2* was not expressed in strain K96243 is similar to the results reported by Ooi et al.^18^. However, *plc2* is expressed in *B. pseudomallei* strain 22^18^, and the Plc2 protein was detected in *B. pseudomallei* strain MSHR668^15^ culture supernatant. These findings indicate that *plc2* gene expression is strain-dependent.

### The *B. pseudomallei plc* genes are induced in infected macrophages

We next investigated expression of the *plc* genes in macrophages. J774A.1 macrophage-like cells were infected with *B. pseudomallei* K96243. At 2, 4, and 6 h post-infection (p.i.), the cells were lysed, and mRNAs corresponding to the *plc* gene detected using RT-PCR. The results showed no expression of the *plc1*, *plc2* or *plc3* genes at 2 h p.i. The expression of all of the *plc* genes was detected at 4 h p.i., but the level of expression of *plc2* was relatively higher than the levels of expression of *plc1* and *plc3.* At 6 h p.i., the levels of expression of all *plc* genes increased compared with 4 p.i. (Fig. 1b). Our finding that the expression of *plc2* gene was induced in macrophages, even though we could not detect expression in culture medium (Fig. 1a), suggests a role of Plc2 in the infection of macrophages.

We stained infected macrophages for LAMP-1 and *B. pseudomallei* using probes labelled with Alexa Fluor 488 or Alexa Fluor 568 and nuclei were stained with DAPI (Fig. 1c). This revealed that at 2 h p.i. most *B. pseudomallei* K96243 were trapped within the phagosome. However, at 4 h p.i, co-localization of *B. pseudomallei* with lysosomes was rare indicating bacterial escape from phagosomes, and almost all of the *B. pseudomallei* cells were not associated with the phagosome at 6 h p.i. It is possible that the intracellular environment induces expression of *plc1, plc2* and *plc3* genes.

Several previous reports show the expression of *plc* genes of other species of bacteria in host tissues^12,13^. For example, the *Mycobacterium tuberculosis plc* genes are up-regulated in macrophages, and the Plcs are cytotoxic to mouse macrophages^7,19^. The *Clostridium perfringens* PLC (α-toxin) is produced in host tissues and can activate the arachidonic acid cascade in cells, with consequent modulation of host immune responses^20^, and the induction of ERK1/2 pathway, resulting in cytotoxicity^21^.

### *B. pseudomallei plc2*, but not *plc1* or *plc3*, is required for bacterial survival and replication in macrophages

**Figure 2:**
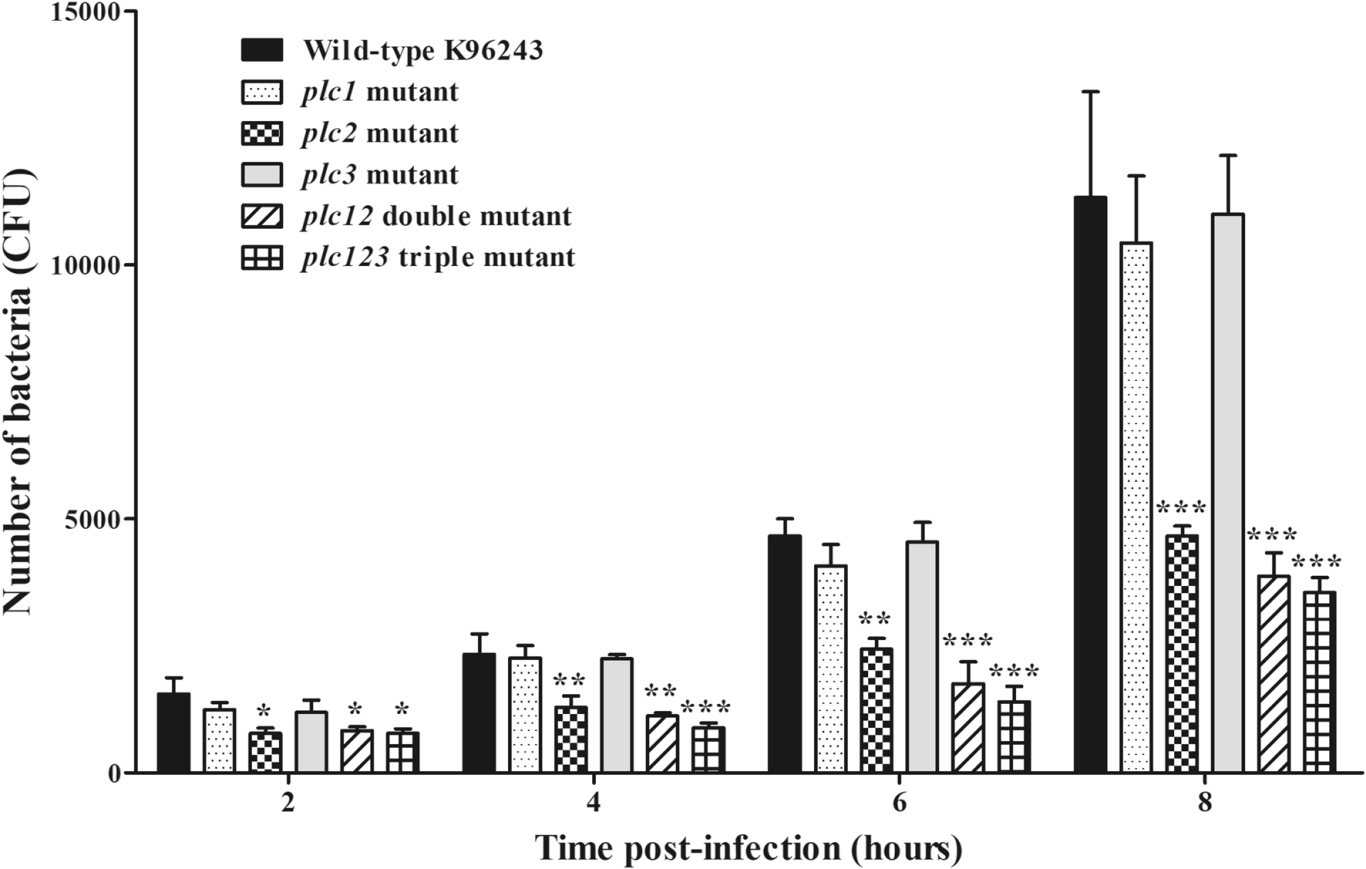
Intracellular growth of *B. pseudomallei* wild-type or *plc* mutants in J774A.1 macrophage-like cells. *B. pseudomallei* wild-type, *plc1*, *plc2*, *plc3*, *plc12* or *plc123* mutants were used to infect J774A.1 cells at an MOI of 0.5. At 2, 4, 6 or 8 h p.i., the infected cells were lysed and bacteria enumerated after plating onto agar. Error bars represent standard error of mean for data collected from 3 independent experiments. Asterisks indicate significant differences (*P < 0.05, **P < 0.01, and ***P < 0.001, one-way ANOVA followed by Dunnett's post hoc test) between *B. pseudomallei* wild-type K96243 and mutant strains. The figure was prepared using GraphPad Prism version 7.05 for Windows (www.graphpad.com).

To provide insight into the role of *plc1, plc2* and *plc3* genes in virulence, we tested a range of single and multiple mutants. We have previously constructed *plc1*, *plc2* and *plc12* mutants^14^ and these were included in our study. Additionally, for this study we constructed *plc3* single and *plc123* mutants by insertion mutagenesis^14^. Mutagenesis of the *plc* genes was confirmed by Southern blotting (data not shown). J774A.1 macrophage-like cells were infected with the mutants. At 2, 4, 6 and 8 h p.i., the numbers of recoverable *plc2*, *plc12* or *plc123* mutants were significantly lower (*P < 0.05, **P < 0.01, ***P < 0.001) than the number of viable wild-type bacteria (Fig. 2). In contrast, there was no significant difference (*P* > 0.05) in the numbers of viable single *plc1* or *plc3* mutants compared with the number of viable wild-type bacteria at all tested time points (2, 4, 6 or 8 h p.i.). These findings indicate that *plc2* gene was required for *B. pseudomallei* survival and replication inside the macrophage. Our results also indicate that *plc1* and *plc3* play no role in survival and replication in macrophages, but we cannot discount the possibility of functional redundancy between these enzymes, which would mask the phenotype associated with the single *plc1* and *plc3* mutants.

**Figure 3:**
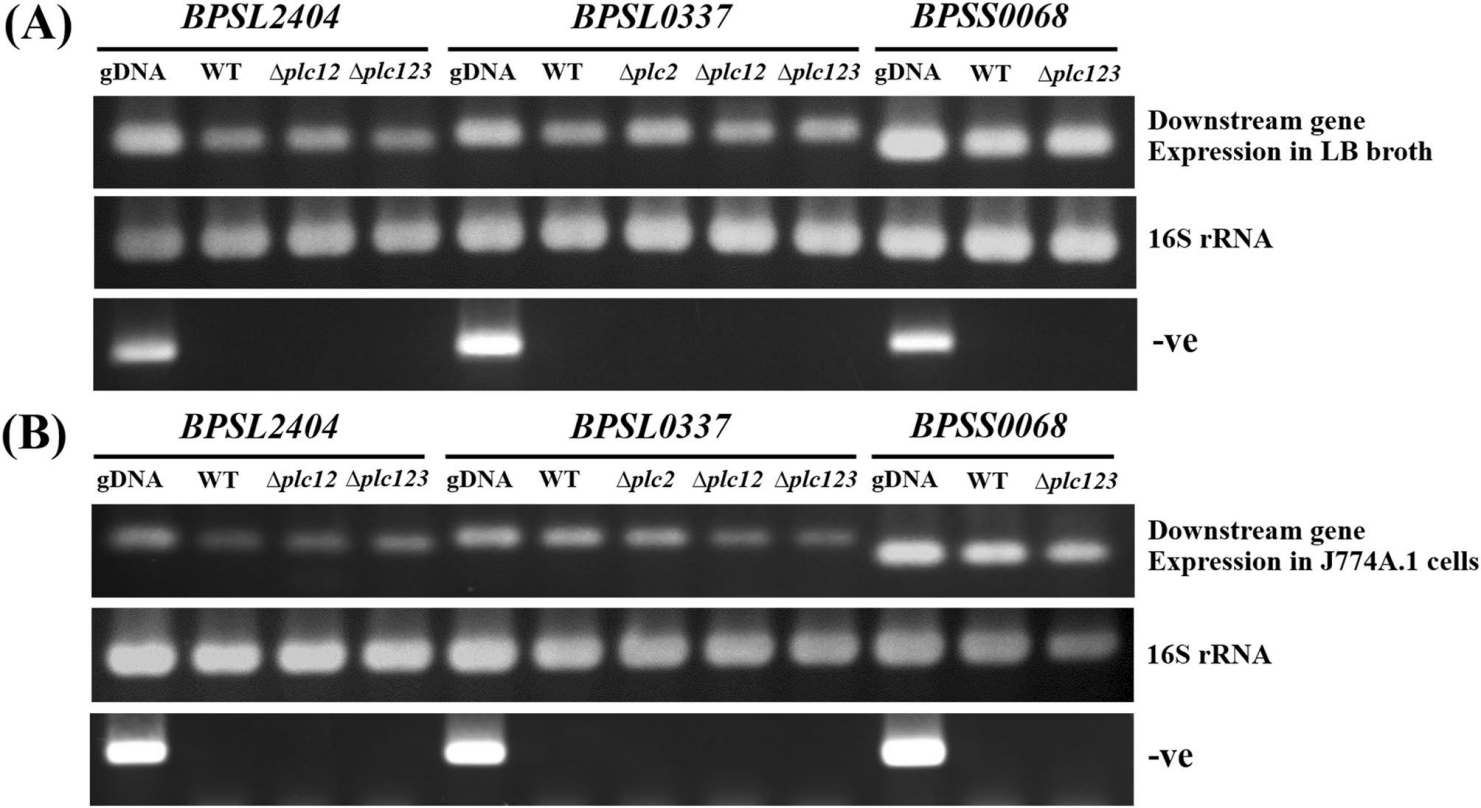
Expression of genes downstream of *plc1, plc2* or *plc3*, assessed using RT-PCR. The mRNA from wild-type, *plc1*, *plc2*, *plc3*, *plc12*, or *plc123* mutants cultured in LB broth (**a**), or isolated from infected J774A.1 macrophages (**b**) was extracted before converting to cDNA as outlined in material and methods (full-length gels are presented in Supplementary Fig. 2). The cDNA was amplified using PCR primers specific to the *bpsl2404*, *bpsl0337* or *bpss0068* genes which are downstream of *plc1*, *plc2* or *plc3*, respectively (upper panel). The 16S rRNA (middle panel) and DNase-treated mRNA (lower panel) were included as a normalization control and negative control, respectively.

To investigate whether the phenotypes we observed with the *plc* mutants was due to polar effects on downstream genes we measured expression of the genes downstream of *plc1*, *plc2* or *plc3* (*bpsl2404*, *bpsl0337* or *bpss0068* respectively) using RT-PCR. *B. pseudomallei* wild-type and the *plc* mutants were cultured in LB broth, or extracted from intracellular bacteria after macrophage infection. We demonstrated similar *bpsl2404*, *bpsl0337* and *bpss0068* amplicons with mRNA from wild-type or *plc1, plc2*, or *plc3* mutants cultured in LB broth (Fig. 3a) and extracted from intracellular bacteria (Fig. 3b) (full-length gels are presented in Supplementary Fig. 2). These results indicate that the insertional mutation in the *plc1*, *plc2*, or *plc3* genes did not abolish the expression of downstream genes. However, we cannot discount the possibility that expression of downstream genes was affected.

### The defect in intracellular survival of the *plc2* mutant is not due to delayed escape from the phagolysosome

**Figure 4:**
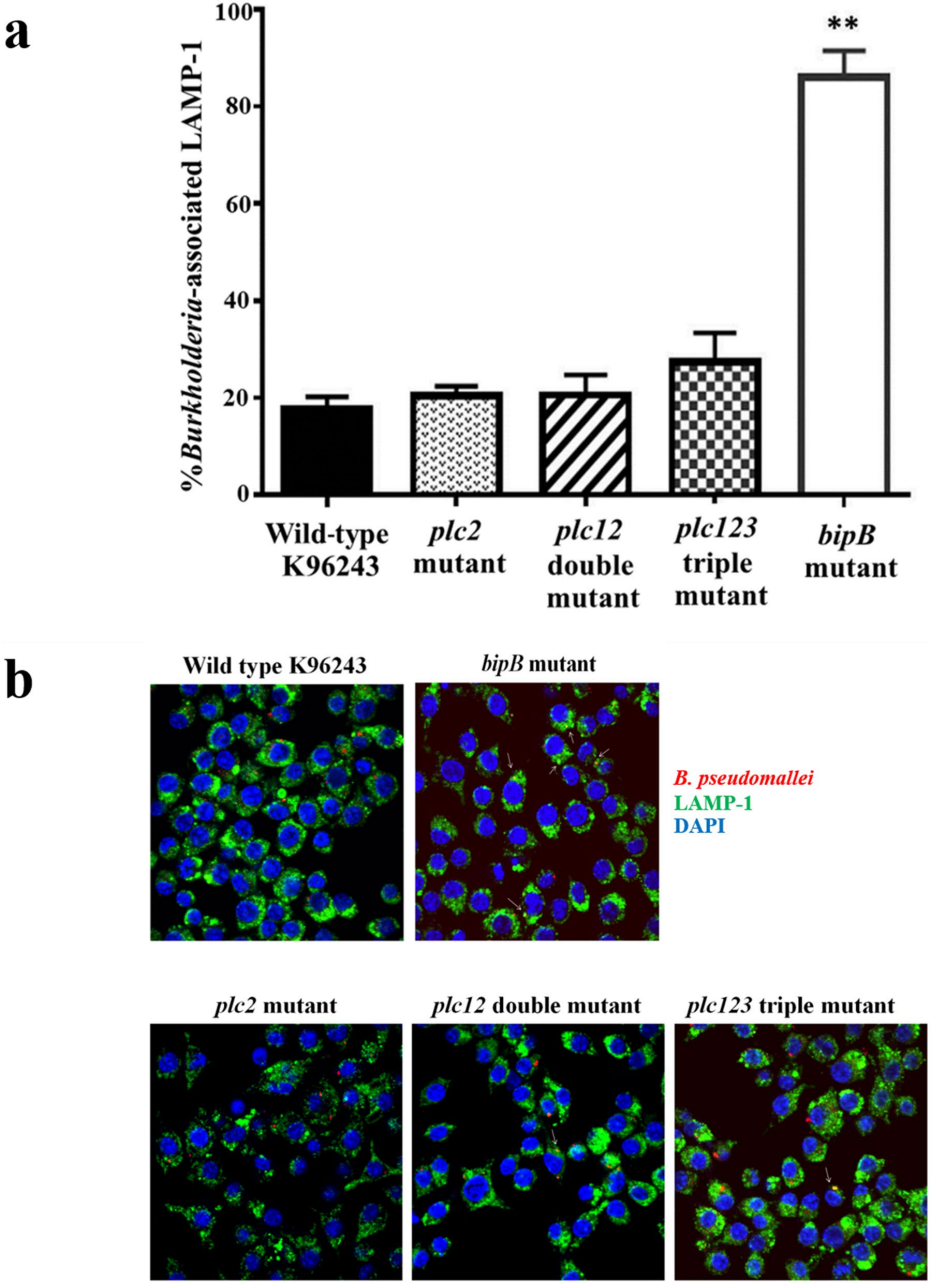
Quantitative analysis of bacterial co-localization with LAMP-1 in J774A.1 macrophage-like cells. (**a**) J774A.1 cells were infected with *B. pseudomallei* wild-type K96243, *plc2*, *plc12*, *plc123* or *bipB* mutants for 3 h. Cells were fixed, permeabilized and immunostained with rat anti-LAMP1 and mouse anti-*Burkholderia* monoclonal antibodies. The y-axis shows the number of bacteria co-localizing with LAMP-1 × 100/total number of intracellular bacteria. Error bars represent standard error of the mean for data collected from 3 independent experiments. Asterisks indicate significant differences (*P* < 0.01*,* students *t*-test) between *B. pseudomallei* wild-type K96243 and mutants. The figure was prepared using GraphPad Prism version 7.05 for Windows (www.graphpad.com). (**b**) Representative confocal micrographs of the association of *B. pseudomallei* with endocytic vesicles in J774A.1 cells. *B. pseudomallei* wild-type K96243, *plc2*, *plc12* double, *plc123* triple or *bipB* mutants were used to infect J774A.1 cells. At 3 h p.i., the infected macrophages and the bacteria were stained and visualised using confocal microscopy. Macrophage LAMP-1 was stained green with rat monoclonal antibody (1D4B) and Alexa Fluor 488 goat anti-rat IgG antibody, and nuclei were stained blue with DAPI. Bacteria were stained red with mouse anti-*B. pseudomallei* monoclonal antibody (9D5) and Alexa Fluor 568 goat anti-mouse IgG antibody. White arrows indicated the co-localization of *B. pseudomallei* and LAMP-1.

The ability of *B. pseudomallei* to escape from the phagosome thought to be considered to be a mechanism by which the bacteria evade phagocyte killing. We had already showed that the *plc1, plc2* and *plc3* genes are expressed in macrophages (Fig. 1c) and that the *B. pseudomallei plc2* mutant was defective in survival in macrophages (Fig. 2). Here we investigated whether Plc2 enzyme played a role in escape from the phagolyosome. J774A.1 macrophage-like cells were infected with either *B. pseudomallei* wild-type, *plc2*, *plc12*, or *plc123* mutants, and co-localization with lysosomes at 3 h p.i. was investigated by immunostaining with antibodies specific to LAMP-1. A *B. pseudomallei bip*B mutant, which is known to be delayed in phagosome escape^22,23^, was included as a control in our experiments. As expected, we found that the majority of *bipB* mutant cells showed delayed escape from phagosome as evidence the increased association with LAMP-1 (86 ± 3.3% association) when compared with the wild-type bacteria (Fig. 4a). The *B. pseudomallei plc2*, *plc12* and *plc123* mutants rarely co-localized with LAMP-1 (20.3 ± 1.2%, 20.7 ± 2.3%, and 27.3 ± 3.5% co-localization, respectively; Fig. 4b), similar to the degree of co-localization of the wild-type bacteria with LAMP-1 (17.7 ± 1.5% co-localization). This finding suggests that mutation of the *plc* genes did not affect escape of *B. pseudomallei* from phagosome.

### *B. pseudomallei plc2* mutant shows deficiency in plaque formation

**Figure 5:**
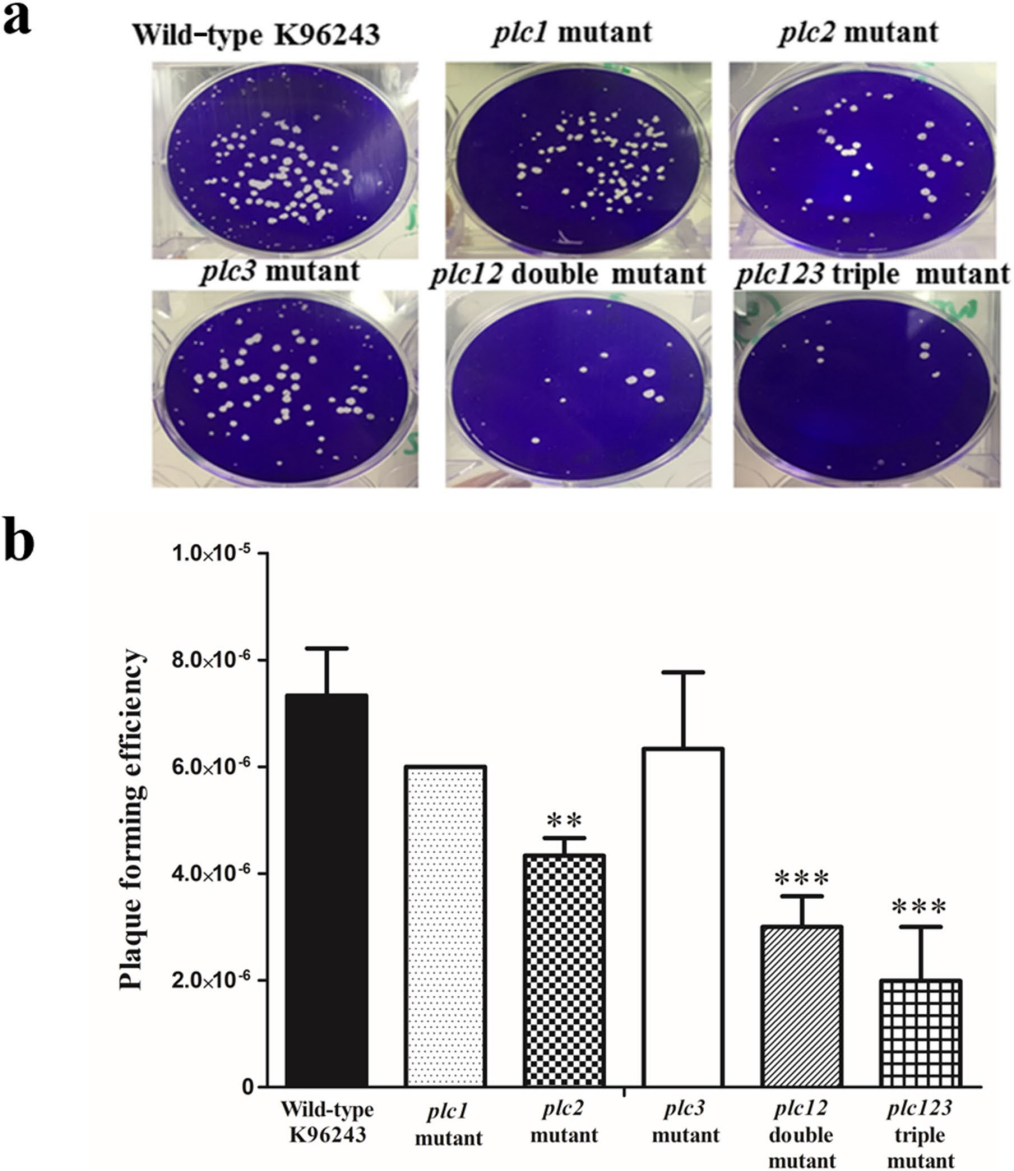
Plaque-forming efficiencies of *B. pseudomallei* wild-type or *plc* mutants in HeLa cells. (**a**) Representative images of plaques. (**b**) Plaque-forming efficiency of either *B. pseudomallei* wild-type K96243, *plc1*, *plc2*, *plc3*, *plc12* or *plc123* mutants in HeLa cells infected at an MOI of 10. Plaques were visualized by crystal violet staining of the monolayers at 24 h p.i. Error bars represent standard error of the mean for data collected in 3 independent experiments. Asterisks indicate significant differences (**P < 0.01, and ***P < 0.001, one-way ANOVA followed by Dunnett's post hoc test) between *B. pseudomallei* wild-type K96243 and mutant strains. The figure was prepared using GraphPad Prism version 7.05 for Windows (www.graphpad.com).

We next measured plaque formation in monolayers of HeLa cells infected with either *B. pseudomallei* wild-type or the *plc1 plc2*, *plc3, plc12* or *plc123* mutants. Plaque-formation reflects the ability of bacteria to invade, survive within and then spread from cell to cell. As shown in Fig. 5, plaque-formation in HeLa cells was significantly reduced in cells infected with the *plc2*, *plc12* or *plc123* mutants, compared to the wild-type strain (**P < 0.01, ***P < 0.001, ***P < 0.001, respectively). In contrast, there was no significant reduction in plaque formation after infection with the *plc1* or *plc3* mutants (*P* > 0 0.05) (Fig. 5). This result shows correlation with our previous study^14^ which showed that plaque-formation efficiency in HeLa cells was significantly reduced after infection with *plc2* or *plc12* double mutants compared to the wild-type strain. Plaque-formation was restored in a *plc2* complemented strain. This finding suggested that the defective phenotype was due to the *plc2* gene mutation^14^. However, complementation of *plc12* double and *plc123* triple mutants was not possible because of restrictions on the use of multiple antibiotic resistance markers in *B. pseudomallei*.

**Figure 6:**
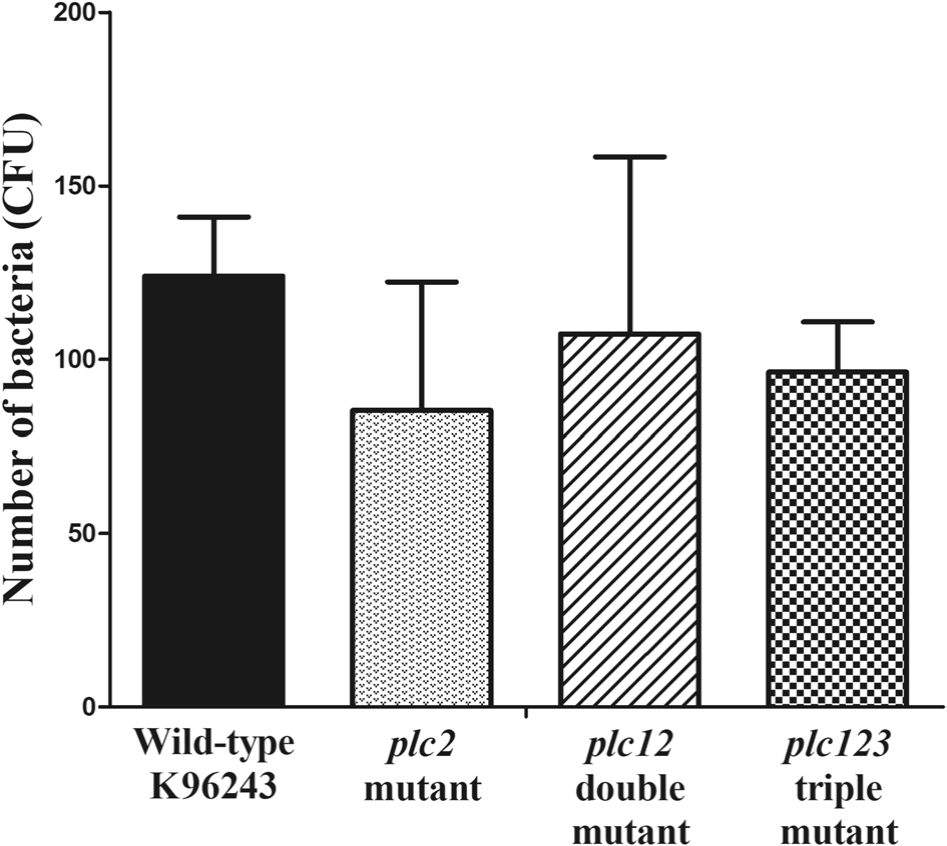
Invasion efficiency of *B. pseudomallei* wild-type and *plc* mutants in HeLa cells. The *B. pseudomallei* wild-type, *plc2*, *plc12* or *plc123* mutants were used to infect HeLa cells at an MOI of 50. At 2 h p.i., the infected cells were lysed and the numbers of intracellular bacteria were enumerated by colony count. Error bars represent standard error of mean for data collected from 3 independent experiments. No significant difference between *B. pseudomallei* wild-type K96243 and mutant strains was detected (*P* > 0.05, one-way ANOVA followed by Dunnett's post hoc test). The figure was prepared using GraphPad Prism version 7.05 for Windows (www.graphpad.com).

To assess whether the reduction in plaque formation reflects a reduced ability to adhere to or to invade HeLa cells, we assessed invasion efficiency. There was no significant difference (*P* > 0 0.05) in the number of culturable intracellular bacteria at 2 h p.i. between wild-type and either *plc2*, *plc12* double*,* or *plc123* triple mutants (Fig. 6). This finding indicates that the absence of the *plc 2* gene had no effect of the bacteria to adhere to or invade HeLa cells. Overall, our findings indicate that Plc2 is required for survival and replication of *B. pseudomallei* in non-phagocytic cells.

### The *plc12* and *plc123* mutants are attenuated in* G. mellonella*

Our results above showed that Plc2 was required for intracellular survival and replication in host cells. To investigate the roles of Plc1, Plc2 and Plc3 in virulence of *B. pseudomallei,* a *G. mellonella* larvae infection model was used^24^.

**Figure 7:**
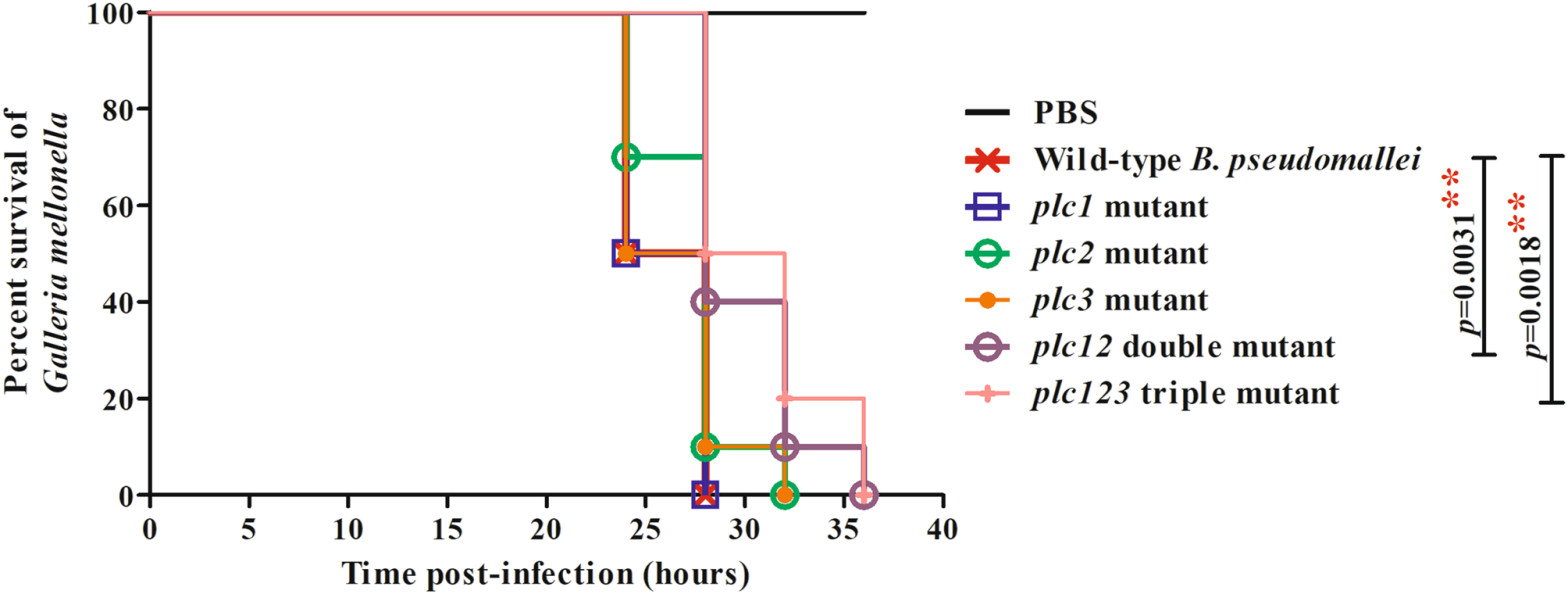
Virulence of *B. pseudomallei* strains in *G. mellonella* larvae. Groups of 10 larvae were challenged with 10^2^ CFU of *B. pseudomallei* wild-type, *plc1*, *plc2*, *plc3*, *plc12* or *plc123* mutants. The number of dead larvae was scored at 24, 28, 32 and 36 h p.i.. GraphPad Prism version 7.05 for Windows (www.graphpad.com) was used to graph and analyze the data using a Log-rank (Mantel-Cox) test. Asterisks indicate significant differences (*P* < 0.05), between *B. pseudomallei* wild-type K96243 and mutant strains. The figure was prepared using GraphPad Prism version 7.05 for Windows (www.graphpad.com).

There was no significant difference (*P* > 0 0.05) in the survival of larvae infected with the *plc1*, *plc2* or *plc3* mutants compared with the wild-type strain (Fig. 7). However, larvae infected with the *plc12* or *plc123* mutants showed significantly (*P* = 0.0031, *P* = 0.0018. respectively) increased survival, compared to larvae infected with the wild-type strain. This finding suggests redundancy of the functions of the phospholipases in virulence of *B. pseudomallei*. Our finding that there was no significant difference (*P* > 0 0.05) in the survival of larvae infected with the *plc12* and *plc123* mutants suggests that *plc3* does not contribute to virulence in *G. mellonella* larvae. Because of the restrictions on the number of antibiotic markers we could introduce into *B. pseudomallei*, we could not generate *plc12* or *plc123* complemented mutants further validate our findings.

Our finding contrasts with a previous study where a *plc3* mutant was found to 10^4^-fold attenuated Syrian hamsters compared to the wild-type bacterium^16^. This might reflect differences in the immune system between *G. mellonella* and mammals. *G. mellonella* possess an innate immune system which involves a cellular immune response mediated by hemocytes, and a humoral immune response orchestrated by antimicrobial peptides^25^, However, they lack the complement system found in mammals and *G. mellonella* also lacks an adaptive immune system^26^. Additionally, it is known that *B. pseudomallei* infection of hamsters is not similar to infection of other mammals such as mice. Hamsters are highly susceptible to infection with *B. pseudomallei* whereas mice are relatively resistant^27^. There are also reports of the different behaviour of *B. pseudomallei* mutants in hamsters and in mice. For example *fliC*^28^ and *fliD*^16^ mutants are not attenuated in hamsters but a *fliC* mutant is highly attenuated in mice^29^.

## Conclusion

The genes encoding three *B. pseudomallei* Plc enzymes are expressed within macrophage-like cells, but at different expression levels. The *plc2* gene was expressed in infected macrophages but not in culture medium, suggesting a role in virulence. Our findings suggest that *plc2* either alone, or in combination with *plc1* and *plc3*, contributes to growth in host cells and our finding that virulence in *G. mellonella* was dependent on the inactivation of genes encoding combinations of Plc enzymes, indicates functional redundancy. The data reported in this study provide important new insight into the roles of Plcs in virulence of *B. pseudomallei* and open new opportunities for further research into the roles on these enzymes in virulence.

## Materials and methods

### Primers, bacterial strains and cell lines

Primers used in this study are shown in Table 1. *Escherichia coli*, *B. pseudomallei* K96243 and the mutant strains were routinely cultured in Luria–Bertani (LB) or trypticase soy medium. *B. pseudomallei* K96243 *plc1*, *plc2* single and *plc12* double mutants were constructed in the previous study^14^. All cultures were typically grown for 24–48 h at 37 °C. Appropriate antibiotics (Sigma-Aldrich) i.e. chloramphenicol 50 µg/mL, kanamycin 400 µg/mL and tetracycline 50 µg/mL were added into the medium if required. All manipulations of *B. pseudomallei* were approved by the Technical Biosafety Committee (TBC), National Center for Genetic Engineering and Biotechnology (BIOTEC).

**Table 1:** Oligonucleotide primers used in this study.

Primers	Oligonucleotide sequences (5′–3′)	Purposes	Sources
PLC88	AGACCGTGCTGCTCGTGAA	Forward primer for construction of *plc3* mutant	This study
PLC89	GGCTCGTTGTTCGGTCGCA	Reverse primer for construction of *plc3* mutant	This study
Plc1F	TGATGCAGGAAAACCGCTC	Forward primer for internal fragment of *plc1* gene	This study
Plc1R	AGCCCGTCCACATGTAGTAG	Reverse primer for internal fragment of *plc1* gene	This study
Plc2F	GCTCGACAACAGCGATTACG	Forward primer for internal fragment of *plc2* gene	This study
Plc2R	TTCTGCAGGATGTTCGTCCC	Reverse primer for internal fragment of *plc2* gene	This study
Plc3F	TCAAGGAAGACATCCGTGCG	Forward primer for internal fragment of *plc3* gene	This study
Plc3R	CGTCGAAATTCACGAGCAGC	Reverse primer for internal fragment of *plc3* gene	This study
23s F	TTTCCCGCTTAGATGCTTT	Forward primer for internal fragment of *23s* RNA gene	^33^
23s R	AAAGGTACTCTGGGGATAA	Reverse primer for internal fragment of *23s* RNA gene	^33^
16s F	AGACACGGCCCAGACTCCTAC	Forward primer for internal fragment of *16s* RNA gene	^33^
16s R	CAGTCACCAATGCAGTTCCCA	Reverse primer for internal fragment of *16s* RNA gene	^33^
*bpsl2404*F-173	GGCAAGGATCTGCAAAACGG	Forward primer for amplification of *bpsl2404* gene	This study
*bpsl2404*R-173	ACGACCGACACCTTCTTGTC	Reverse primer for amplification of *bpsl2404* gene	This study
*bpsl0337*F-184	TCCCCAGTTCCTCCTCGATT	Forward primer for amplification of *bpsl0337 *gene	This study
*bpsl0337*R-184	ATGCAACACACCGAACAACC	Reverse primer for amplification of *bpsl0337* gene	This study
*bpss0068*F-158	CTGCCGATGCCGGATTATCA	Forward primer for amplification of *bpss0068* gene	This study
*bpss0068*R-158	AACGAATTTGCTTGCTCGGG	Reverse primer for amplification of *bpss0068* gene	This study

J774A.1 murine macrophage-like and human epithelial HeLa cells were obtained from the American Type Culture Collection (ATCC) and were cultured in Dulbecco’s Modified Eagle medium supplemented with 10% (v/v) heat-inactivated fetal bovine serum (Invitrogen) under a 5% CO_2_ atmosphere at 37 °C in a humidified incubator.

### RNA preparation and reverse transcription (RT)-PCR analysis

*B. pseudomallei* K96243 was grown in LB broth for 6 h before incubation at 37 °C for 15 min in LB broth pH 4.5, 5.0, 6.0, 7.0, 8.0 or 9.0. Total RNA was extracted from 10^8^ CFUs *B. pseudomallei* cultured in each condition using TRIZOL (Invitrogen) according to manufacturer’s instructions. The isolated bacterial RNA was then treated with DNase I (Ambion) to remove any genomic DNA contamination before use.

To detect *B. pseudomallei* genes expression within macrophages, monolayers of J774A.1 murine macrophage-like cells were infected with the bacteria. At the indicated time points, the infected cell monolayers were washed and subsequently lysed with 500 μL of 0.1% Triton X-100 (Sigma-Aldrich) to allow intracellular bacteria released from infected cells. Then, 500 μL of 1 × PBS was added and the intracellular bacterial RNA were extracted using TRIZOL (Invitrogen) according to manufacturer’s instructions.

To convert the extracted total RNA to cDNA, SuperScript III First-Strand Synthesis System (Invitrogen) was used. The cDNA was quantified and adjusted so that similar quantities were included in the PCR reactions. The cDNA was amplified using the PCR with primers (Table 1), GoTaq DNA polymerase (Promega) and cycling conditions of 94 °C, 3 min and 30 cycles of 94 °C for 30 s, 50 °C for 1 min, and 72 °C for 45 s, followed by incubation at 72 °C for 5 min. In each PCR experiment, the amplification of 23S rRNA was used as a normalization control.

### Construction of *B. pseudomallei plc3* single and *plc123* triple mutants

A *B. pseudomallei plc3* mutant was constructed by insertion mutagenesis^30^. A 406-bp (nucleotide positions 1052–1457) internal region of the *plc3* gene was amplified from *B. pseudomallei* K96243 genomic DNA with primers PLC88 and PLC89 (Table 1). The amplified DNA fragment was ligated into *Eco*RV digested pKNOCK-Cm, a suicide vector^30^ to generate recombinant plasmid pVSK3 for insertion mutagenesis. The constructed *plc3* mutant was selected on *Pseudomonas* agar base supplemented with SR103E (Oxoid) and chloramphenicol.

To construct the *B. pseudomallei plc123* triple mutant, the amplified internal *plc3* fragment (nucleotide positions 1052-1457) was ligated into *Eco*RV digested pKNOCK-Km^30^. This constructed plasmid, designated pVSK4, was introduced into *B. pseudomallei plc12* double mutant^14^. The mutants were selected on *Pseudomonas* agar base (Oxoid) supplemented with SR103E (Oxoid) containing chloramphenicol, kanamycin and tetracycline (Sigma-Aldrich). The *plc123* mutant was verified by PCR and Southern blotting.

### Intracellular survival and plaque assays

Intracellular replication of *B. pseudomallei* in macrophage-like cells was assessed as described previously^31^ with some modifications. Briefly, J774A.1 murine macrophage-like cells were seeded at a density of 2.5 × 10^5^ cells per well of a 24-well tissue culture plate and infected approximately 24 h later with *B. pseudomallei* wild-type K96243 or *plc* mutant at a multiplicity of infection (MOI) of approximately 0.5, for 2 h. Then infected cells were overlaid with DMEM medium (Invitrogen) containing gentamicin 128 μg/mL and spectinomycin 256 μg/mL (Sigma-Aldrich) to kill extracellular bacteria. The infected cell monolayers were subsequently lysed at 2, 4, 6 and 8 h p.i. with 0.1% Triton X-100 (Sigma-Aldrich). The numbers of intracellular bacteria were quantified by serial dilution and plating on tryptic soy agar. Bacterial colony forming units (CFU) were counted after 36–48 h of incubation at 37 °C. Plaque forming assays were performed as described previously^32^. The plaque-forming efficiency was calculated as the number of plaques/bacterial CFU added per well.

### Confocal analysis of bacterial co-localization with LAMP-1

The intracellular localizations of *B. pseudomallei* wild-type K96243 and the *plc* mutants in J774A.1 macrophages cells relative to LAMP-1 containing vesicles were investigated according to previously described^33^. Briefly, Macrophages were infected for 2 h at a multiplicity of infection (MOI) of 2 and incubated at 37 °C, 5% CO_2_. At different time points, *B. pseudomallei* infected J774A.1 cells were fixed in 4% paraformaldehyde, the monolayers were permeabilized with 0.5% (v/v) Triton X-100, and blocked with 1% (w/v) bovine serum albumin. Bacteria were detected with a 1:10 dilution of mouse anti-*Burkholderia* monoclonal antibody and detected with a 1:200 dilution of Alexa Fluor 568-goat anti-mouse IgG (Invitrogen, USA). LAMP1 was stained green with a 1:100 dilution of rat monoclonal antibody (1D4B; Abcam, USA) and 1:500 Alexa Fluor 488 goat anti-rat IgG antibody (Molecular Probes, USA), and nuclei were stained blue with a 1:500 dilution of 4,6-diamidino-2-phenylindole (DAPI). Cells were examined by a laser-scanning confocal microscope equipped with LSM5 Image Browser (LSM 510 META, Carl Zeiss, Germany). The association of *Burkholderia* with LAMP1 was considered when the red fluorescent bacteria co-localized with the green fluorescence of LAMP1-positive vacuoles, represented as an area of yellow staining.

The percentage of intracellular *Burkholderia* associated with LAMP-1 was determined as the number of bacteria co-localized with LAMP-1/total number of intracellular bacteria × 100. For the quantitative analysis of the association of intracellular *B. pseudomallei* strains with LAMP-1 containing vesicles, at least 200 individual bacteria associated LAMP-1 containing vesicles were monitored.

### Virulence in *G. mellonella*

Virulence in *G. mellonella* larvae was tested as described previously^24^ with some modifications. Larvae between 2–2.5 cm and free of melanization or injury were used in the experiments. To prepare the bacterial culture for infection, the overnight cultures of *B. pseudomallei* wild-type and the mutants were adjusted to a concentration of 10^4^ CFUs per ml in PBS. A 701 N fixed needle syringe (Hamilton, Nevada) was used to inject 10 µl aliquots of the bacterial suspension into the *G. mellonella* larvae to get the final concentration of 10^2^ CFUs. Injections were performed directly into the larval body cavity and groups of 10 larvae were injected with each bacterial strain. Control larvae were injected with PBS. Following injection, larvae were incubated in the dark at 37 °C and the number of dead larvae were recorded at a variety of times post injection. The *Galleria mellonella* study was approved by the Mahidol University-Institute Animal Care and Use Committee (U1-05763-2559).

### Statistical analysis

For in vivo mutant characterization, a log-rank (Mantel-Cox) test was used to compare survival curves and the experiments for comparison between groups were performed using the one-way ANOVA followed by Dunnett's post hoc test within the GraphPad Prism version 7.05 for Windows (www.graphpad.com, GraphPad Software, CA, USA). *P*-values less than 0.05 were considered statistically significant (*P < 0.05, **P < 0.01, and ***P < 0.001).

## Supplementary information

Supplementary Information.
