# Supplementary material for: Functional redundancy of Burkholderia pseudomallei phospholipase C enzymes and their role in virulence
Source: Sci Rep. 2020 Nov 6;10:19242. doi: 10.1038/s41598-020-76186-z (PMC7648637; doi:10.1038/s41598-020-76186-z)
Supplement: Supplementary file 1 — Supplementary Information. [file 41598_2020_76186_MOESM1_ESM.docx]

**Supplementary information**

**Functional Redundancy of *Burkholderia pseudomallei* Phospholipase C Enzymes and Their Role in Virulence**

Varintip Srinon^1,2¶^, Patoo Withatanung^1¶^, Somjit Chaiwattanarungruengpaisan^3^, Metawee Thongdee^3^, Chatruthai Meethai^1^, Joanne M. Stevens^4^, Richard W. Titball^5^, & Sunee Korbsrisate^1^*

^1^Department of Immunology, Faculty of Medicine Siriraj Hospital, Mahidol University, Bangkok, Thailand. ^2^Veterinary Diagnostic Center, Faculty of Veterinary Science, Mahidol University, Nakhon Pathom, Thailand. ^3^The Monitoring Surveillance Center for Zoonotic Diseases in Wildlife and Exotic Animals, Faculty of Veterinary Science, Mahidol University, Nakhon Pathom, Thailand. ^4^Division of Infection and Immunity, The Roslin Institute and Royal (Dick) School of Veterinary Studies, University of Edinburgh, Midlothian, United Kingdom. ^5^Department of Biosciences, University of Exeter, Exeter**,** United Kingdom.

^¶^These authors contributed equally: Varintip Srinon and Patoo Withatanung

*Correspondence and requests for materials should be addressed to SK (email: Sunee.kor@mahidol.edu)

**Short title:** Redundancy of *Burkholderia pseudomallei* phospholipase C enzymes

**Supplementary figure 1**

Reverse transcription (RT)-PCR analysis of *B. pseudomallei plc* expression. The mRNA from *B. pseudomallei* cultured in LB broth adjusted to pH 4.5, 5, 7, 8 or 9 was extracted before converting to cDNA as outlined in material and methods. The cDNA was amplified using PCR primers specific to the *plc1* (405 bp), *plc2* (174 bp), *plc*3 (186 bp) or *23s rRNA* (334 bp) genes. Positive control (+ve) was *B. pseudomallei* genomic DNA. DNase-treated bacterial RNA was used as a negative control (-ve) to confirm the absence of DNA contamination in RNA samples.

**
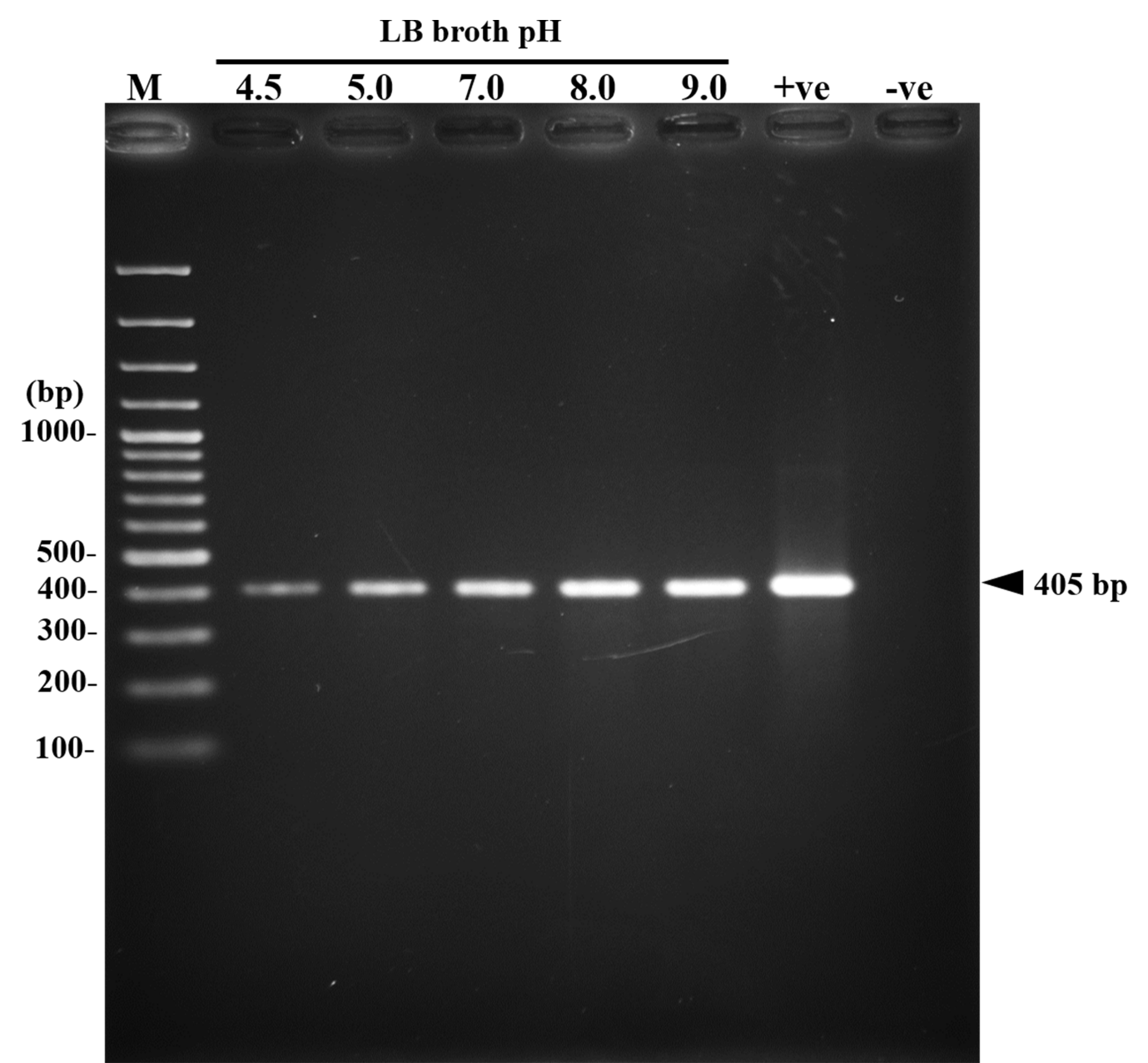
**

**Fig. S1a** The cDNA of *B. pseudomallei* cultured in LB broth adjusted to pH 4.5, 5, 7, 8 or 9 was amplified using PCR primers specific to the ***plc1*** gene (405 bp).

**
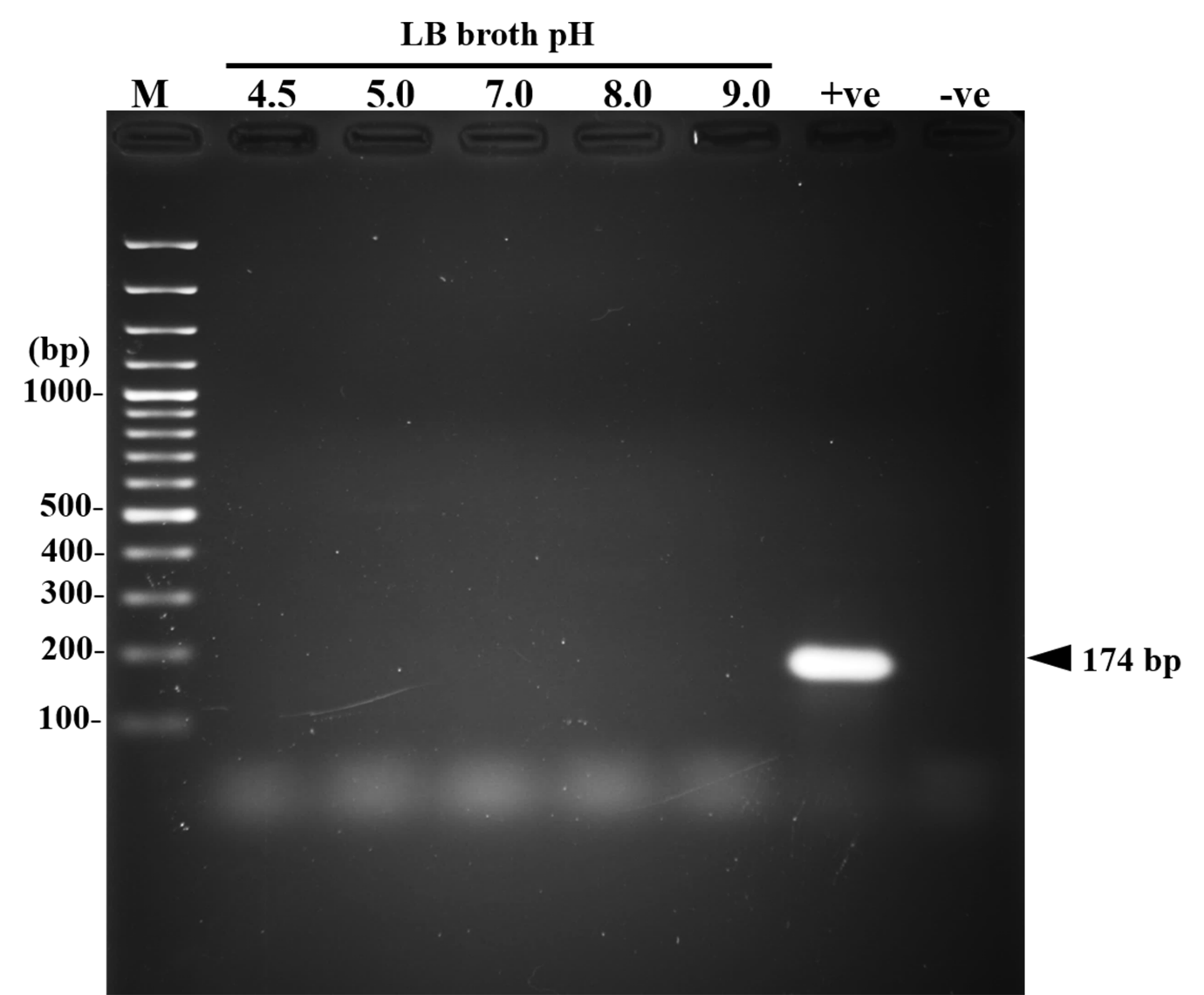
**

**Fig. S1b** The cDNA of *B. pseudomallei* cultured in LB broth adjusted to pH 4.5, 5, 7, 8 or 9 was amplified using PCR primers specific to the ***plc2*** gene (174 bp).

**
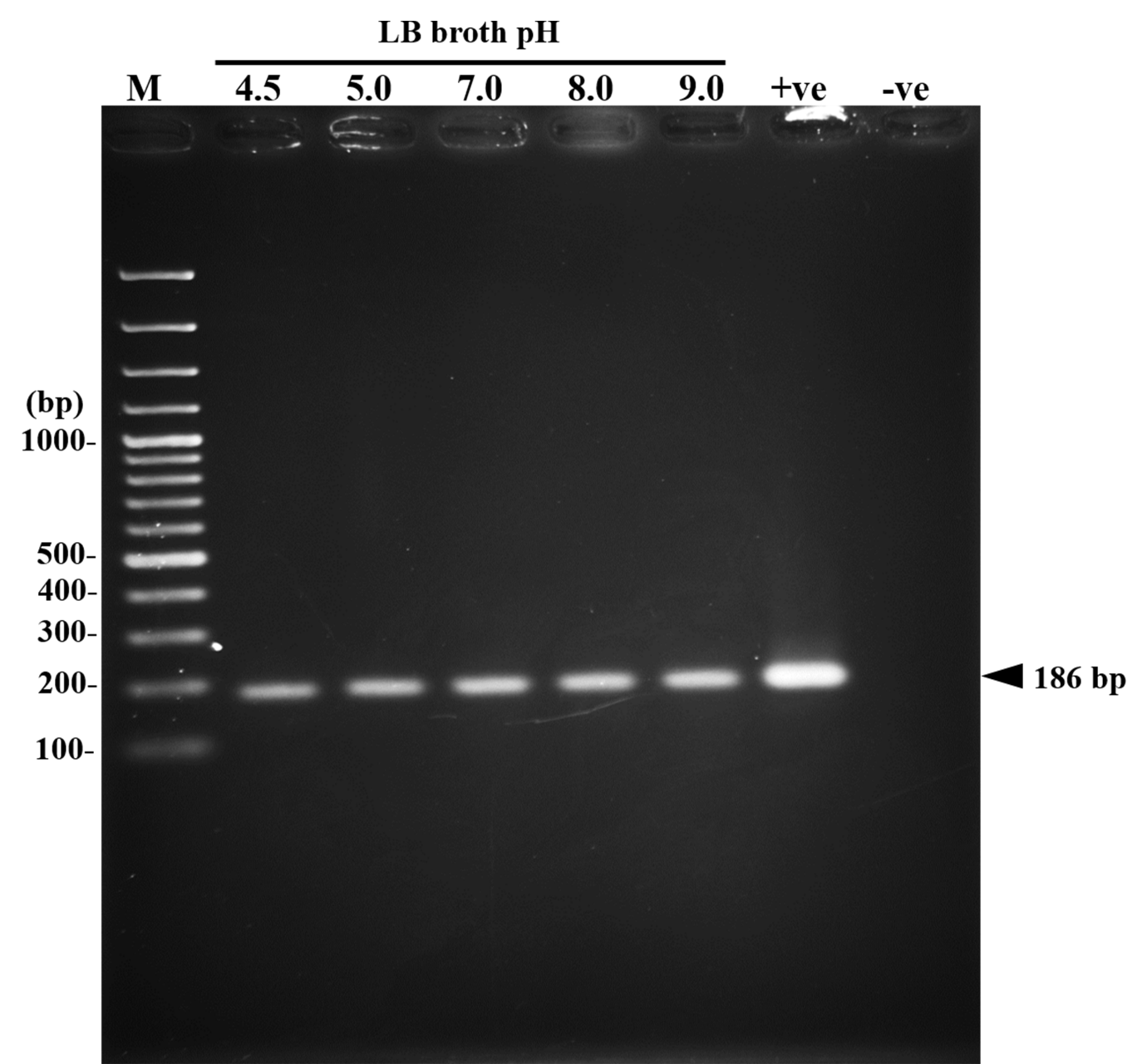
**

**Fig. S1c** The cDNA of *B. pseudomallei* cultured in LB broth adjusted to pH 4.5, 5, 7, 8 or 9 was amplified using PCR primers specific to the ***plc3*** gene (186 bp).

**
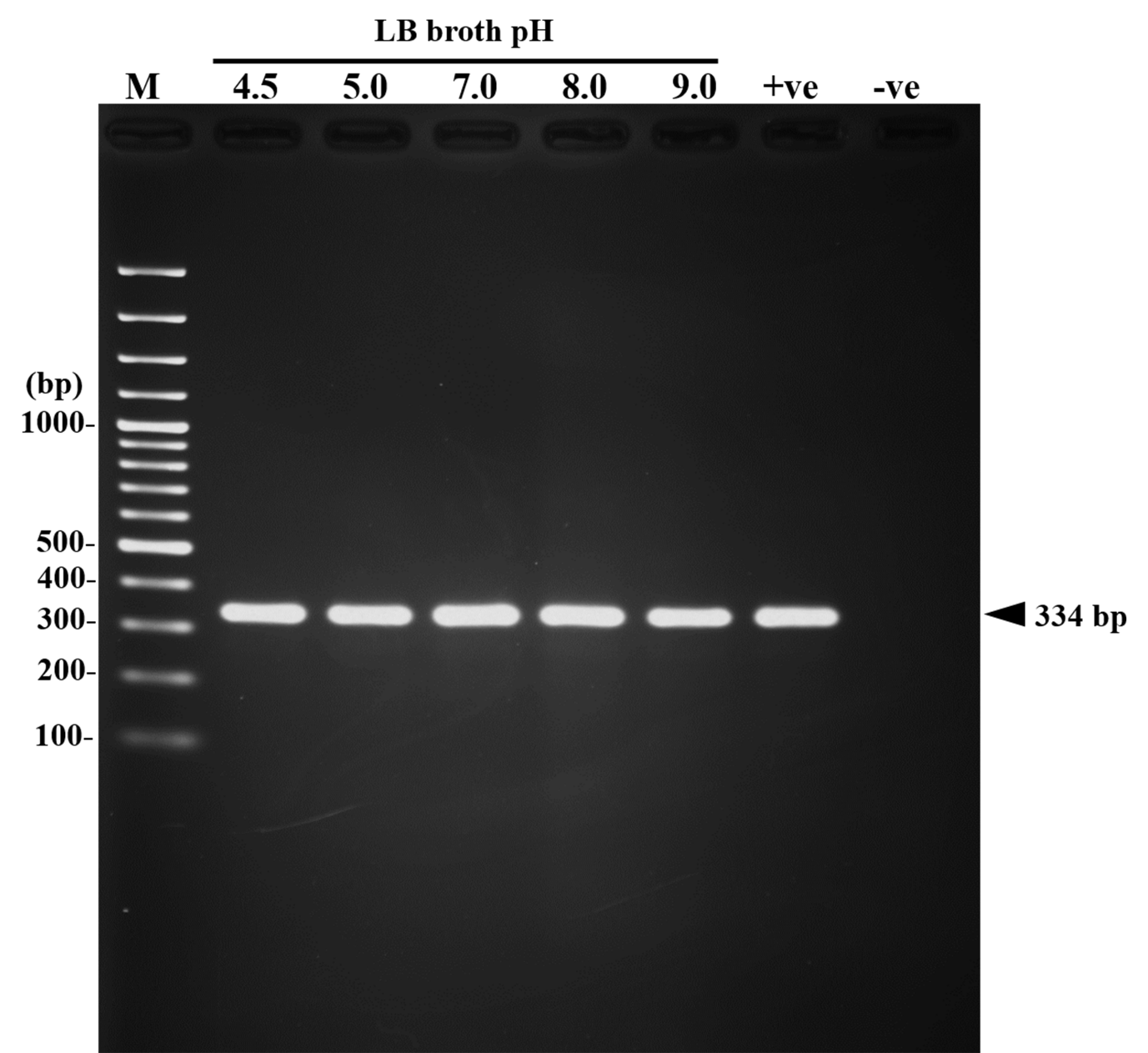
**

**Fig. S1d** The cDNA of *B. pseudomallei* cultured in LB broth adjusted to pH 4.5, 5, 7, 8 or 9 was amplified using PCR primers specific to the ***23s rRNA*** gene (334 bp).

**Supplementary figure 2**

RT-PCR analysis of *B. pseudomallei* *plc1, plc2* and *plc3* downstream genes expressions. The mRNA from *B. pseudomallei* wild-type, *plc1*, *plc2*, *plc3*, *plc12*, or *plc123* mutants cultured in LB broth (Figs. S2a-c), or isolated from infected J774A.1 macrophages (Figs. S2d-f) was extracted before converting to cDNA as outlined in material and methods. The cDNA was amplified using PCR primers specific to the *bpsl2404* (173 bp), *bpsl0337* (184 bp) or *bpss0068* (158 bp) genes which are downstream of *plc1*, *plc2* or *plc3*, respectively (Figs. S2a, d). The 16S rRNA (Figs. S2b, e) and DNase-treated mRNA (Figs. S2c, f) were included as a normalization control and negative control, respectively. These results indicate that there are no polar effects caused by the insertional mutation in the *plc1*, *plc2*, or *plc3* genes.


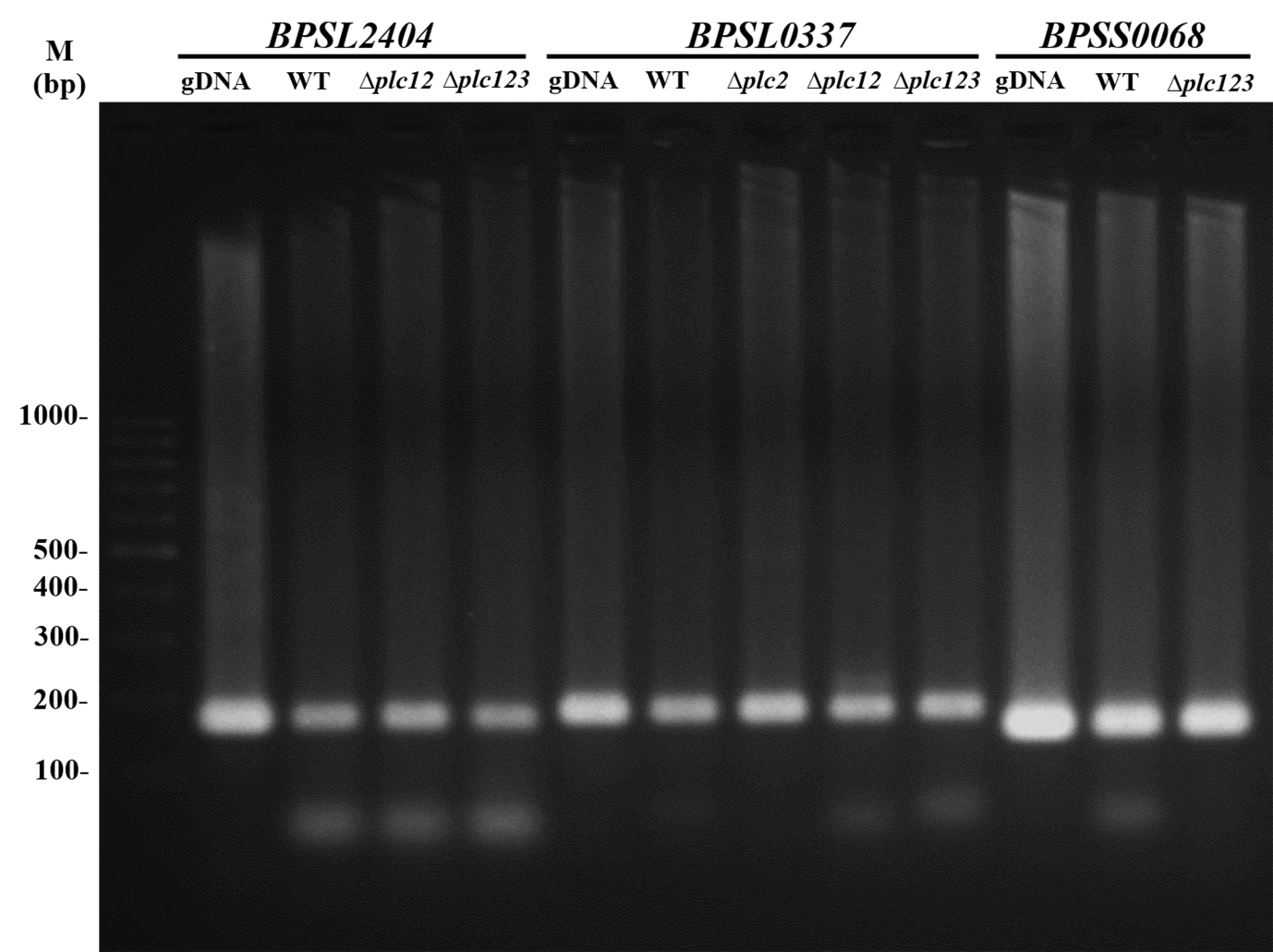


**Fig. S2a** The cDNA of *B. pseudomallei* wild-type, *plc1*, *plc2*, *plc3*, *plc12*, or *plc123* mutants cultured in **LB broth** was amplified using PCR primers specific to the *bpsl2404* (173 bp), *bpsl0337* (184 bp) or *bpss0068* (158 bp) genes which are downstream of *plc1*, *plc2* or *plc3* genes, respectively.


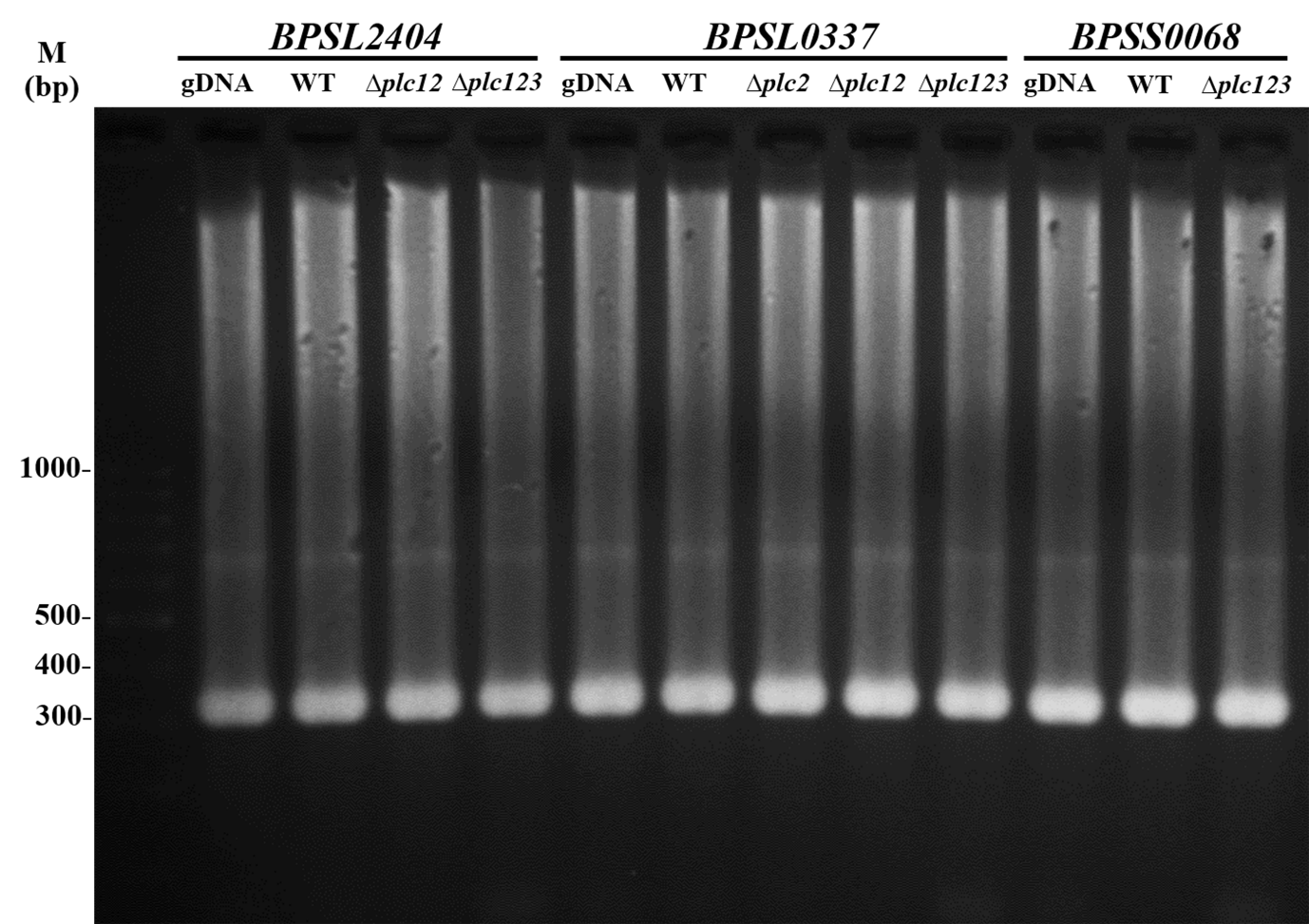


**Fig. S2b** The cDNA of *B. pseudomallei* wild-type *plc1*, *plc2*, *plc3*, *plc12*, or *plc123* mutants cultured in **LB broth** was amplified using PCR primers specific to **16S rRNA** gene as a normalization control.


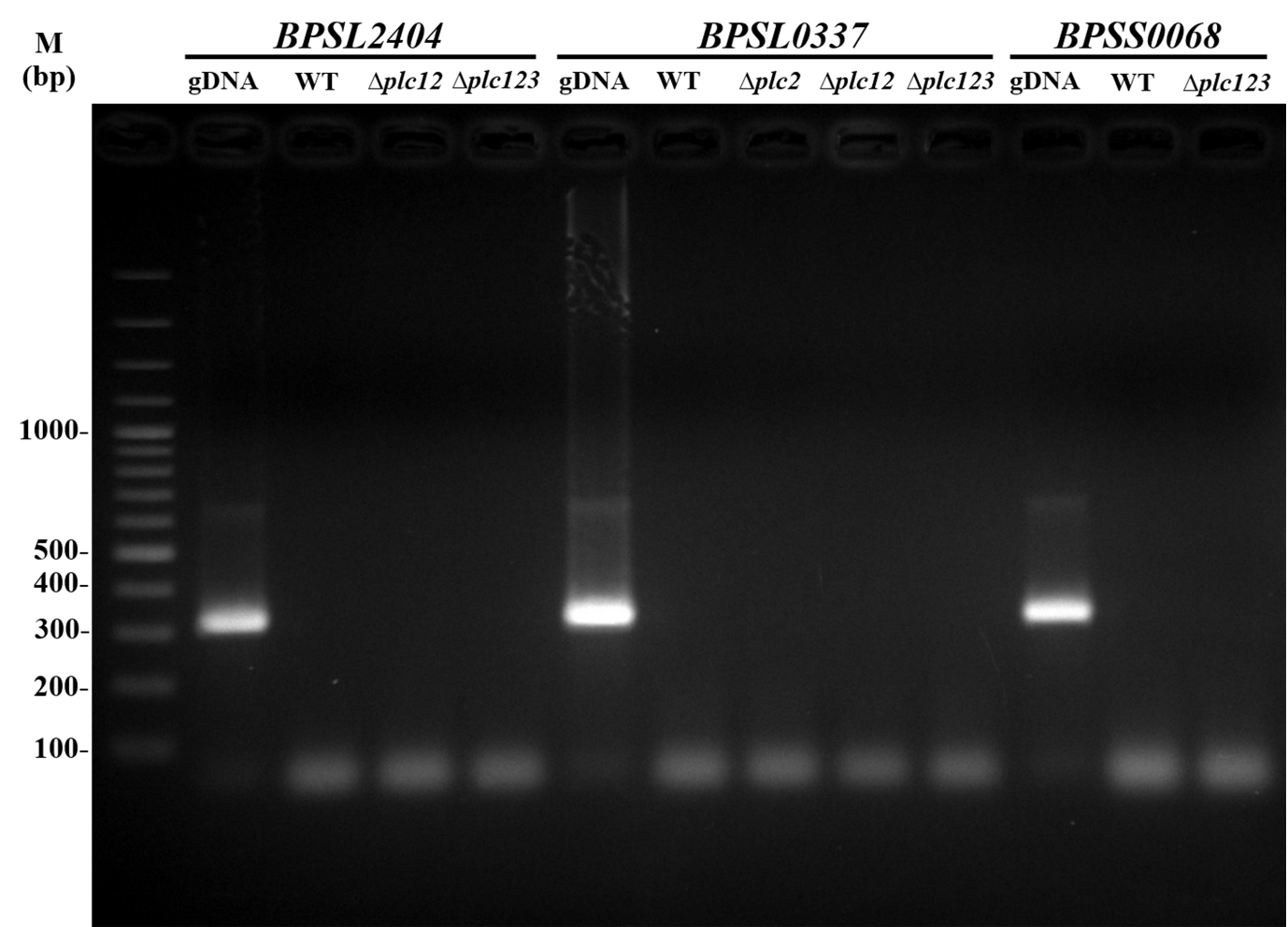


**Fig. S2c** The DNase-treated mRNA of *B. pseudomallei* wild-type, *plc1*, *plc2*, *plc3*, *plc12*, or *plc123* mutants cultured in **LB broth** was amplified using PCR primers specific to 16S rRNA as a negative control. Genomic DNA of *B. pseudomallei* was included as positive control.

**
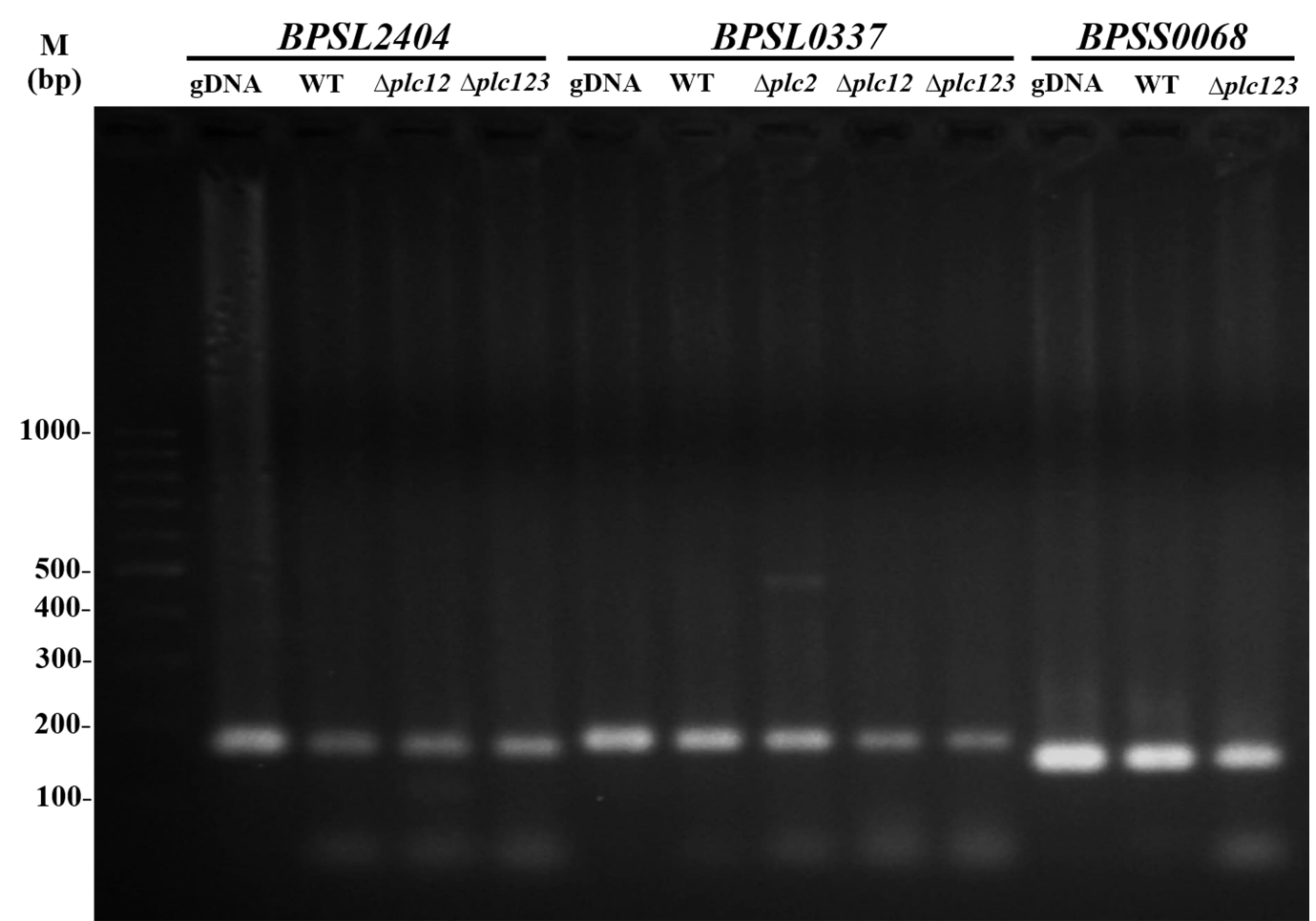
**

**Fig. S2d** The cDNA of *B. pseudomallei* wild-type, *plc1*, *plc2*, *plc3*, *plc12*, or *plc123* mutants isolated from infected **J774A.1 macrophages** was amplified using PCR primers specific to the *bpsl2404* (173 bp), *bpsl0337* (184 bp) or *bpss0068* (158 bp) genes which are downstream of *plc1*, *plc2* or *plc3* genes, respectively.

**
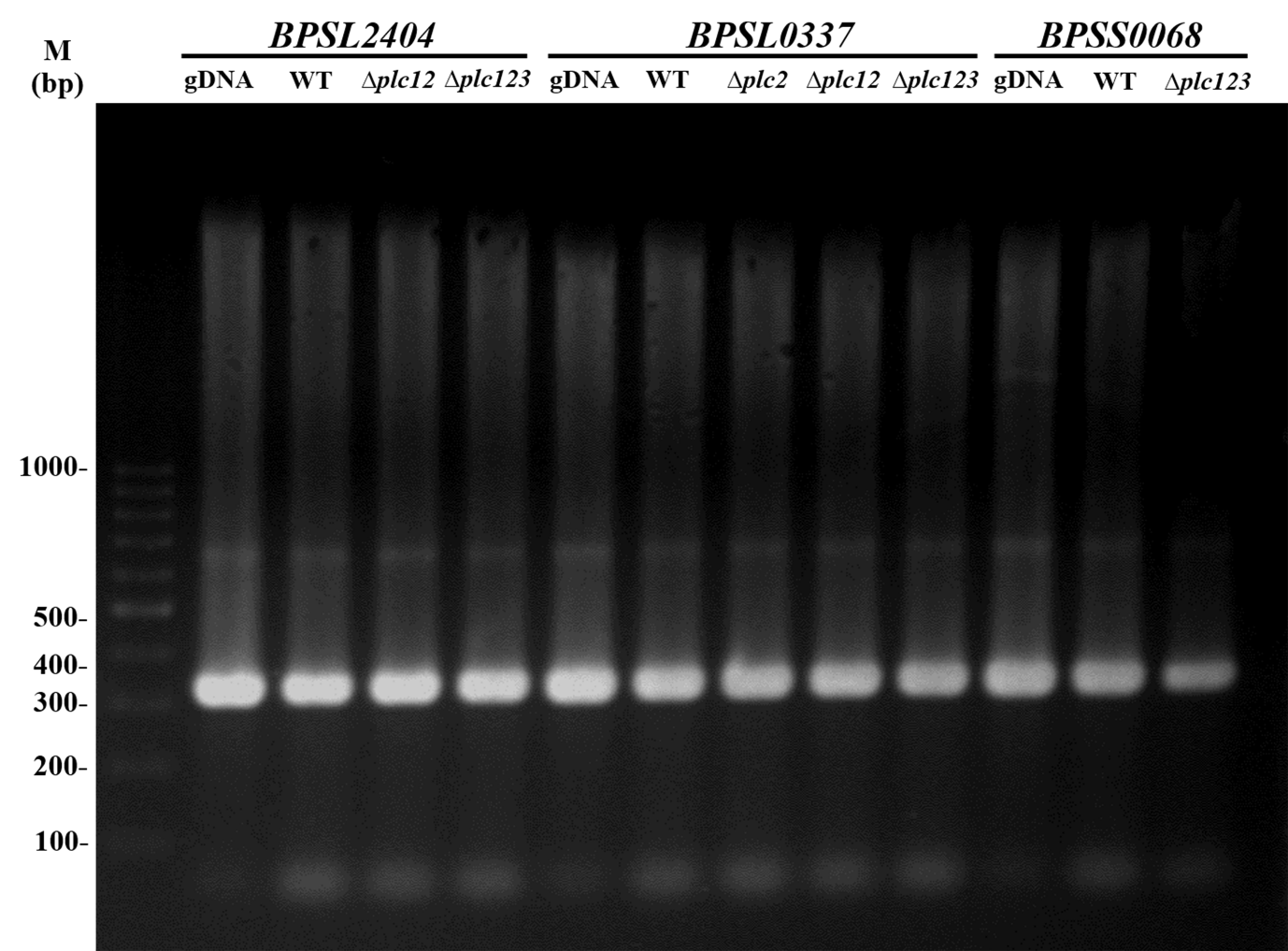
**

**Fig. S2e** The cDNA of *B. pseudomallei* wild-type, *plc1*, *plc2*, *plc3*, *plc12*, or *plc123* mutants isolated from infected **J774A.1 macrophages** was amplified using PCR primers specific to **16S rRNA** gene as a normalization control.


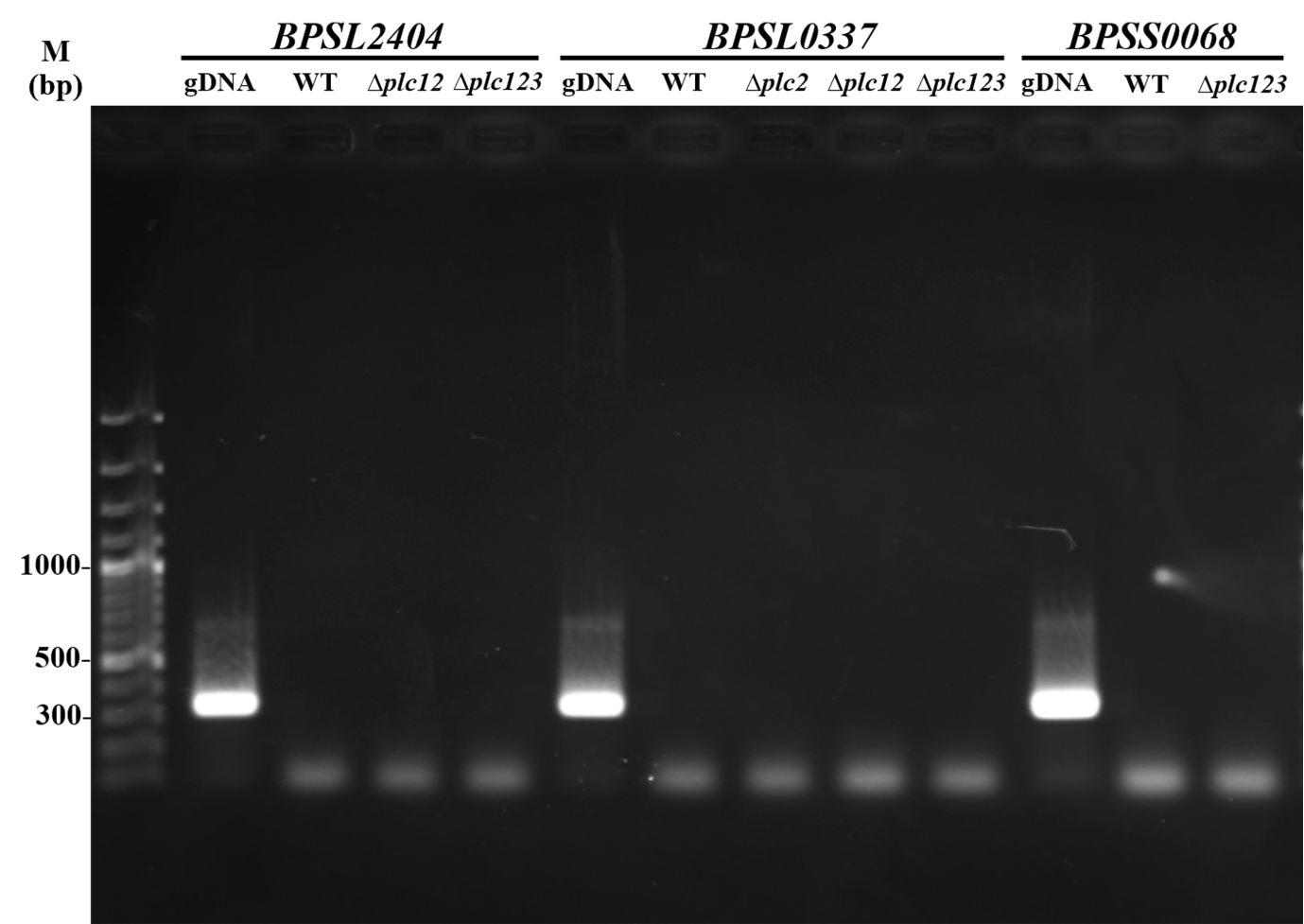


**Fig. S2f** The DNase-treated mRNA of *B. pseudomallei* wild-type, *plc1*, *plc2*, *plc3*, *plc12*, or *plc123* mutants isolated from infected J774A.1 macrophages was amplified using PCR primers specific to 16S rRNA as a negative control. Genomic DNA of *B. pseudomallei* was included as positive control.
